# Antibiotic treatment protocols revisited: the challenges of a conclusive assessment by mathematical modelling

**DOI:** 10.1098/rsif.2021.0308

**Published:** 2021-08-25

**Authors:** Hildegard Uecker, Sebastian Bonhoeffer

**Affiliations:** ^1^ Institute of Integrative Biology, ETH Zurich, Universitätstrasse 16, Zurich 8092, Switzerland; ^2^ Max Planck Institute for Evolutionary Biology, August-Thienemann-Strasse 2, Plön 24306, Germany

**Keywords:** antibiotic resistance, hospital-acquired infections, antimicrobial stewardship, mathematical models

## Abstract

Hospital-acquired bacterial infections lead to prolonged hospital stays and increased mortality. The problem is exacerbated by antibiotic-resistant strains that delay or impede effective treatment. To ensure successful therapy and to manage antibiotic resistance, treatment protocols that draw on several different antibiotics might be used. This includes the administration of drug cocktails to individual patients (combination therapy) but also the random assignment of drugs to different patients (mixing) and a regular switch in the default drug used in the hospital from drug *A* to drug *B* and back (cycling). For more than 20 years, mathematical models have been used to assess the prospects of antibiotic combination therapy, mixing and cycling. But while tendencies in their ranking across studies have emerged, the picture remains surprisingly inconclusive and incomplete. In this article, we review existing modelling studies and demonstrate by means of examples how methodological factors complicate the emergence of a consistent picture. These factors include the choice of the criterion by which the effects of the protocols are compared, the model implementation and its analysis. We thereafter discuss how progress can be made and suggest future modelling directions.

## 1. Introduction

For many decades, bacterial infections have been successfully treated with antibiotics, making formerly life-threatening diseases easily treatable. However, the rapid evolution of resistance and the slow discovery of new antimicrobial compounds increasingly reduce treatment options. In the European Union, resistant bacteria are responsible for more than 33 000 deaths per year, as estimated based on data from 2015 [1]. On the one hand, to stop this alarming trend, restrictions in the use of antibiotics are needed. On the other hand, antibiotics must be used as wisely as possible whenever their application is required. Unfortunately, knowing what is wise is far from obvious, and we need to understand what the consequences of different treatment strategies are to be able to make more rational choices.

While they are unable to replace empirical research and clinical trials, mathematical models have helped to gain insight into the effects of antimicrobial stewardship. Mathematical studies profit from several strengths. They rely on explicit and well-defined assumptions, allow us to explore ideas much faster than clinical trials and are not subject to practical and ethical restrictions. A question that has been repeatedly addressed in theoretical studies over the past 20 years concerns the integrated application of multiple antibiotics across a community—usually a hospital ward—during the phase of empirical therapy, i.e. during initial treatment that is administered before the responsible bacterium has been identified (table 1). The idea is that strains that are resistant to one of the drugs are suppressed by another. Within a single patient, this can be achieved by the administration of two (or more) antibiotics in combination (combination therapy). Another multi-drug strategy for the treatment of individual patients is sequential therapy, in which the different antibiotics are alternated instead of being given simultaneously. This strategy has attracted increasing attention in recent years [20–23], but has to our knowledge not been modelled on a hospital scale yet and is therefore not discussed further in this review. Across a community, it is an option to prescribe different drugs to different patients in order to create a heterogeneous environment for the bacteria. Most prominently, the default drug can be cycled in time (cycling), creating temporal heterogeneity, or a fraction of patients can receive each drug (mixing), creating spatial heterogeneity. The use of two (or more) antibiotics in either form, however, comes at the risk of selecting for double (or multiply) resistant strains that can withstand all drugs used. Identifying which strategy best treats infections in the face of resistance and at the same time selects least for multiply resistant bacteria is challenging. In practice, the increased risk of side effects and higher economic costs possibly associated with combination therapy are additional factors but current theoretical work only assesses the disease dynamics and emergence of resistance under the various strategies and omits other aspects.

**Table 1.** Literature overview. The studies listed in the table were found by a general literature search for mathematical models of antibiotic therapy across a community, including following up on the references and citations of the articles found. We excluded studies that only consider one of the strategies without comparing it with at least one of the others. Best strategy: we list the strategy that emerges as the best one overall. A strategy that still seems to be worth noting based on its performance is added in brackets. When the picture as a whole remains inconclusive but a strategy seems to have some advantage over the others, we note this strategy in brackets.

publication	comparison	optimality criterion	best strategy	*R*_*AB*_ compartment	deterministic/ stochastic	memory	analysis
Bonhoeffer *et al.* [2]	CYC, MIX, COMB	— no. of uninfecteds in a given time interval	COMB (MIX; CYC)	yes	det.	no	analytical results and computer simulations of a single parameter set
		— no. of uninfecteds until double resistance has reached 50%^a^	MIX (CYC, COMB)				
		— time until double resistance has reached 50%^a^	inconclusive				
Bergstrom *et al.* [3]	CYC, MIX	— average no. of infecteds with res. strain	MIX (CYC)	no	det.	no	parameter sensitivity analysis
		— evol. of double resistance via horizontal gene transfer	inconclusive				
Levin & Bonten [4]	CYC, MIX	average no. of infecteds with res. strain	MIX	no	det.	no	computer simulations of individual parameter sets
Beardmore & Peña-Miller [5]	reactive CYC^b^, CYC, MIX	— no. of infecteds in a given time interval	reactive CYC	no	det.	no	optimal control theory, computer simulations of a single parameter set
		— no. of infecteds with res. strain	reactive CYC				
Bonhoeffer *et al.* [6]	reactive CYC, CYC, MIX	— no. of infecteds in a given time interval	MIX	no	det.	no	computer simulations of individual parameter sets
		— no. of infecteds with res. strain	MIX				
Beardmore & Peña-Miller [7]	CYC, MIX	— no. of infecteds in a given time interval	inconclusive	no	det.	no	analytical considerations, computer simulations of a single parameter set
		— no. of infecteds with res. strain	inconclusive				
Sun *et al.* [8]	SINGLE, CYC, MIX, COMB	not employed	not discussed	yes	det.	no	analytical treatment of the equilibria
Kouyos *et al.* [9]	CYC, MIX, ISS^c^	— prevalence of resistance	ISS	yes	stoch.	yes	computer simulations of individual parameter sets
		— no. of inappropriately treated patients	ISS				
Chan *et al.* [10]	SINGLE, MIX, COMB, THRESH^d^, DIFF^e^, POC^f^	prevalence of infections in time	inconclusive (MIX)	yes	det.	no	computer simulation of a single parameter set
Obolski & Hadany [11]	CYC, MIX, COMB	— evol. of double resistance via mutation	CYC	no	det.	no	moving average over 10^4^ parameter sets
		— no. of infected patients	COMB				
		— emergence of double resistance via horizontal gene transfer	CYC (MIX)				
Abel zur Wiesch *et al.* [12]	CYC, MIX	combination of no. of inappropriately treateds and no. of symptomatically infecteds	inconclusive (CYC at optimal frequency)	yes	both	yes	parameter sensitivity analysis
Campbell & Chao [13]	NONE, CYC, ‘MIX’^g^, COMB, MONO, CONTROL^h^	average no. of uninfecteds in equilibrium	COMB	yes	det.	no	combination of analytical results and computer simulations of individual parameter sets
Xiridou *et al.* [14]	SINGLE, COMB, THRESH^d^	prevalence of infecteds	COMB	yes	det.	no	computer simulation of 1000 parameter sets
Obolski *et al.* [15]^i,k^	SINGLE, CYC, MIX	— mean no. of incorrectly treated patients	MIX	no	det.	no	computer simulation of a single parameter set
		— evol. of double resistance	inconclusive				
Beardmore *et al.* [16]	CYC, MIX, reactive CYC	— no. of infecteds with res. strain	inconclusive (reactive CYC)	no	stoch.	no	conceptual considerations + stochastic computer simulations of individual parameter sets
		— no. of infected patient days	inconclusive (reactive CYC)				
		— *mean length of hospital stay*^j^	*reactive cycling*	*NA*	*stoch.*	*both versions*	*individual-based computer simulations of individual parameter set for a nested model of within-host and between-host dynamics*						
Tepekule *et al.* [17]	SINGLE, CYC, MIX, COMB, reactive CYC	gain in no. of uninfecteds in 1 year compared with no treatment	COMB (all others)	yes	det.	no	parameter sensitivity analysis
Uecker & Bonhoeffer [18]^k^	CYC, MIX	— average no. of uninfecteds	inconclusive	yes	det.	yes	computer simulations of single parameter sets
		— spread of double resistance	inconclusive				
Houy & Flaig [19]	COMB, CYC, METRO^l^, MONO, THRESH-k^*m*^, INFOBEST^n^	average cumulative number of infected patient days within 2 years^n^	THRES-1	yes	stoch.	no	averaging over 400 parameter sets

^a^These two criteria have not been systematically studied. ^b^Reactive cycling denotes a strategy where the default drug is always the one for which resistance is currently less prevalent. For MIX and CYC, drugs can be used unequally (i.e. different proportions in MIX; different periods of use in CYC), and for both strategies the performances under optimal drug use are compared. ^c^ISS stands for ‘informed switching strategy’. The antibiotic for incoming patients depends on the prevalence of resistance to both drugs in the hospital, and several variants of ISS are tested. The winning strategy is ISS_LAST_. In this strategy, the latest time point at which resistance to either drug is detected determines which drug is used. ^d^For THRESH, drug *A* is used until resistance has reached a threshold. ^e^For DIFF, different strategies are used depending on the risk group, defined through the rate of partner change. ^f^For POC, point-of-care testing is available such that resistant infections can be identified and treated accordingly. ^g^Mixing is different here since each half of the population cycles the drugs. ^h^For CONTROL, evolution of resistance is impossible (we exclude it from the comparison). ^i^The main text of the article focuses on the effect of restricted versus an equal use of a third antibiotic. We only consider the briefly investigated two-drug model from electronic supplementary material, supplementary information S3. ^j^The part in italic letters uses an individual-based nested model of within-host and between-host dynamics. ^k^These two studies do not aim to generally assess the performance; we report the results for the examples shown in the articles. ^l^METRO: Combination therapy is alternated with periods of no drug use. ^m^THRES-*k*: Combination therapy is given if the number of double-resistant infections is below *k*; otherwise, no drugs are administered. ^n^INFOBEST: The best strategy for a given parameter set. This is by definition the best strategy but requires perfect information regarding the parameters. We exclude it from the comparison.

Modelling studies tend to rank the three principal treatment protocols in the order ‘combination therapy > mixing ≥ cycling’ but the picture is not conclusive (table 1). No strategy is optimal under all circumstances [16,17], and to date, despite substantial efforts, it has not been conclusively resolved which conditions favour one or the other strategy. Why is it so difficult to obtain a clear picture? In this article, we pinpoint the difficulties that mathematical studies face in the assessment of antimicrobial treatment protocols. We entirely focus on modelling studies for this review. It should be noted though that clinical studies have not come to definite conclusions either (e.g. [24–28]). Assessing the risk of evolution and spread of resistant strains in clinical trials is hard, in part because of their timelines and in part because of the stochastic nature of the evolutionary dynamics. This makes the importance of gaining clear insights from modelling even more clear.

To illustrate the difficulties faced by theoretical studies, we set up a model for the spread of bacterial infections within a hospital that follows the traditional modelling approach. It combines features of the two original models by Bonhoeffer *et al.* [2] and Bergstrom *et al.* [3], similar to the model by Tepekule *et al.* [17]. We chose to explicitly refer to a hospital setting because the framework is most relevant for antibiotic treatment in hospitals; however, for most of this article, this is only a choice of wording. We apply this model to demonstrate, by means of examples, how the ranking of strategies is affected by factors other than the biology of the pathogen. The most important one is the choice of the optimality criterion by which the performance of a treatment protocol is assessed. Owing to the complexity of the problem, which requires consideration from several aspects, multiple criteria are in use, making study outcomes difficult to compare. But also purely technical aspects can pose obstacles in arriving at congruent conclusions. We discuss how these problems might be addressed and how current models could be extended, helping mathematical models to better meet their potential in assessing antimicrobial treatment protocols.

## 2. The modelling framework

While differing in many respects, almost all existing studies are based on the same general approach. They follow the number of uninfected and infected patients over time, where infected patient populations are divided up into several classes according to the infecting bacterial strain (which is characterized by the resistance profile). For concreteness, we introduce the modelling framework by presenting the model that we apply throughout the article.

The examples in the present article are based on an overarching model that brings together elements from Bonhoeffer *et al.* [2] and Bergstrom *et al.* [3] (as detailed below) and incorporates the most fundamental processes, which are influx and efflux of patients, infection, clearance, the *de novo* emergence of resistance and replacement infection, but it does not incorporate more detailed features such as explicitly modelled drug interactions [17,18]. As in all studies except for the short commentary article by Levin & Bonten [4], we consider the use of two (and not more) drugs. All issues raised in the present article carry over to future models that would incorporate more drugs. (There is no *a priori* reason to assume that the ranking of strategies is independent of the number of drugs used.) The same applies to models considering other strategies such as informed cycling strategies that make use of information on the prevalence of resistance [5,9].

**Figure 1.**
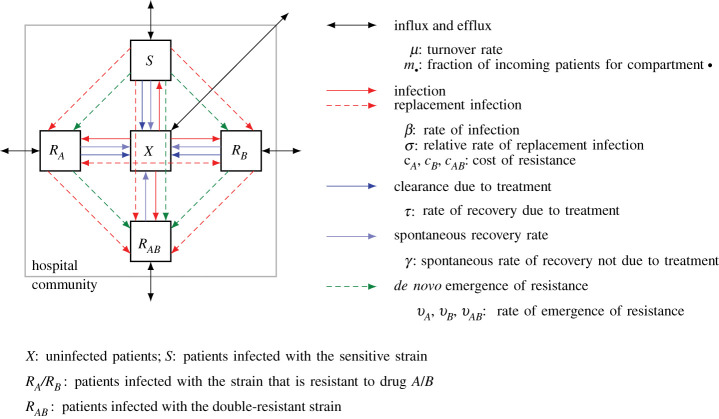
Flow diagram of the model defined by equation (2.1). The diagram shows the five model compartments and the processes that lead to patient flow between them. Solid lines describe epidemiological processes; processes marked by dashed lines involve evolution and competition between strains at the within-host level. Adapted from [17,18].

A flow diagram of the model is shown in figure 1. Patients can be uninfected or infected by one of four bacterial strains—the sensitive strain that responds to both drugs the two strains that are resistant to only one drug, and the double-resistant strain. Following the convention in the field, we denote the number of uninfected patients by *X*, the number of patients infected by the sensitive strain by *S* and the number of patients infected by a resistant strain by *R*_*A*_, *R*_*B*_ and *R*_*AB*_, respectively.

New patients get admitted to the hospital at a total rate of *μn*_tot_, where *n*_tot_ is the total number of patients in the hospital. They can be uninfected or infected with one of the strains as given by the probability $m_{∙}$ (with ‘∙’ standing for *X*, *S*, *A*, *B* or *AB* and indicating the compartment). Irrespective of infection status, patients leave the unit at a *per capita* rate *μ*, i.e. the bacterial infection neither increases mortality nor requirese stationary treatment. *μ* is thus the turnover rate. Patients can get newly infected within the hospital. The transmission rate for the sensitive strain is *β*. The cost of resistance manifests itself in a lower transmission rate (reduction by factors (1 − *c*_*A*_), (1 − *c*_*B*_) and (1 − *c*_*AB*_), respectively). While we exclude co-infection from the model and assume that every patient is only colonized by a single strain at any time, we allow for the instantaneous replacement of infecting strains by better-adapted strains, which we term ‘replacement infection’. Colonization of an infected patient happens at a lower probability than colonization of an uninfected patient (reduction by a factor *σ*). The immune system clears infections at rate *γ*. A drug to which the infecting strain is susceptible leads to recovery at rate *τ*.

Finally, resistance can evolve under drug pressure. During treatment with drug *A* or *B*, resistance to the respective drug evolves at rate *ν*_*A*_ and *ν*_*B*_, respectively. Sensitive strains become resistant to both drugs simultaneously at rate *ν*_*AB*_. These rates combine mutation and fixation of the resistant strain; they also contain selection of pre-existing mutants. We assume that reversal of drug resistance within a patient due to back mutation or replacement infection through the sensitive strain is negligible.

We assume that only infected patients receive antibiotics (no prophylactic treatment). *χ*_*A*_, *χ*_*B*_ and *χ*_*AB*_ are the fractions of infected patients that get treated with drug *A*, drug *B* or both drugs, respectively. As done in most modelling studies, we assume that the treatment protocols can be perfectly implemented. For combination therapy, we thus have *χ*_*AB*_ = 1; for mixing, *χ*_*A*_ = *χ*_*B*_ = 1/2; for cycling, *χ*_*A*_ = 1, *χ*_*B*_ = 0 in periods during which drug *A* is used and *χ*_*A*_ = 0, *χ*_*B*_ = 1 in periods during which drug *B* is used. We always start the cycling protocol with drug *A*. Note that these fractions are identical for all compartments and constant in time (except for drug cycling).

Overall, we obtain the following set of ordinary differential equations (ODEs) that describe the flow between the different compartments:

2.1 $$\begin{array}{rl} & \frac{dS}{dt}=m_{S}n_{\mathrm{tot}}\mu -\mu S-(\gamma +\tau )S-(\chi_{A}\nu_{A}+\chi_{B}\nu_{B}+\nu_{AB})S+\beta SX\\ & \;-\chi_{A}\sigma (1-c_{A})\beta SR_{A}-\chi_{B}\sigma (1-c_{B})\beta SR_{B}-(1-c_{AB})\sigma \beta SR_{AB},\\ & \frac{dR_{A}}{dt}=m_{A}n_{\mathrm{tot}}\mu -\mu R_{A}-(\gamma +(\chi_{B}+\chi_{AB})\tau )R_{A}-(\chi_{B}+\chi_{AB})\nu_{B}R_{A}\\ & \;+\beta (1-c_{A})R_{A}X+\chi_{A}\nu_{A}S+\chi_{A}\sigma \beta (1-c_{A})SR_{A}\\ & \;+\sigma \beta \{\chi_{A}(1-c_{A})-\chi_{B}(1-c_{B})\}R_{A}R_{B}-(\chi_{B}+\chi_{AB})\sigma \beta (1-c_{AB})R_{A}R_{AB},\\ & \frac{dR_{B}}{dt}=m_{B}n_{\mathrm{tot}}\mu -\mu R_{B}-(\gamma +(\chi_{A}+\chi_{AB})\tau )R_{B}-(\chi_{A}+\chi_{AB})\nu_{A}R_{B}\\ & \;+\beta (1-c_{B})R_{B}X+\chi_{B}\nu_{B}S+\chi_{B}\sigma \beta (1-c_{B})SR_{B}\\ & \;-\sigma \beta \{\chi_{A}(1-c_{A})-\chi_{B}(1-c_{B})\}R_{A}R_{B}-(\chi_{A}+\chi_{AB})\sigma \beta (1-c_{AB})R_{B}R_{AB},\\ & \frac{dR_{AB}}{dt}=m_{AB}n_{\mathrm{tot}}\mu -\mu R_{AB}-\gamma R_{AB}+\nu_{AB}S+(\chi_{B}+\chi_{AB})\nu_{B}R_{A}\\ & \;+(\chi_{A}+\chi_{AB})\nu_{A}R_{B}+\beta (1-c_{AB})R_{AB}X+\sigma \beta (1-c_{AB})\sigma SR_{AB}\\ & \;+(\chi_{B}+\chi_{AB})\sigma \beta (1-c_{AB})R_{A}R_{AB}+(\chi_{A}+\chi_{AB})\sigma \beta (1-c_{AB})R_{B}R_{AB}\\ \mathrm{and}\;\;\;\; & \frac{dX}{dt}=(1-m_{S}-m_{A}-m_{B}-m_{AB})n_{\mathrm{tot}}\mu -\mu X+(\gamma +\tau )S-\beta SX+(\gamma +(\chi_{B}+\chi_{AB})\tau )R_{A}\\ & \;-\beta (1-c_{A})R_{A}X+(\gamma +(\chi_{A}+\chi_{AB})\tau )R_{B}-\beta (1-c_{B})R_{B}X+\gamma R_{AB}-\beta (1-c_{AB})R_{AB}X. \end{array}\}$$

We numerically integrate equation (2.1) using Mathematica version 10.4.1.0 (Wolfram Research).

To conclude the outline of the model, we briefly give some details on the choice of model in the context of the existing literature. Our model combines features of the models by Bonhoeffer *et al.* [2] and Bergstrom *et al.* [3], which were among the first studies published. Bonhoeffer *et al.* [2] do not specifically refer to antibiotic treatment in hospitals but generally across a community. There is only influx into the compartment of uninfecteds (in our model that would mean *m*_*S*_ = *m*_*A*_ = *m*_*B*_ = *m*_*AB*_ = 0). Resistance may pre-exist (as reflected by the initial conditions of the system) or emerge *de novo* during treatment. Unlike in our model, the cost of resistance is implemented as a higher pathogen recovery rate rather than a reduced pathogen transmission rate. The model in Bonhoeffer *et al.* [2] allows for a higher death rate of infecteds compared with uninfecteds, while the efflux rate *μ* is the same for all patients in our model. Bonhoeffer *et al.* [2] assume that the protocols can be perfectly implemented such that all patients are treated according to the respective treatment protocol, and we make this assumption as well. The study by Bergstrom *et al.* [3] explicitly refers to a hospital setting. In contrast to Bonhoeffer *et al.* [2] and our model, their ODE system does not contain the *R*_*AB*_ compartment. Double resistance is studied by considering the rate at which it first appears. For example based on our model, under combination therapy, double resistance appears at rate *ν*_*A*_*R*_*B*_(*t*) + *ν*_*B*_*R*_*A*_(*t*) + *ν*_*AB*_*R*_*AB*_(*t*). We discuss this model implementation in more detail below. Bergstrom *et al.* [3] allow for influx from the outside into all patient classes, and we allow for this as well. The cost of resistance is implemented as a lower rate of transmission. There is no *de novo* emergence of resistance contained in their model equations. Efflux rates are the same for all patients, as in our model. Bergstrom *et al.* [3] include the possibility that some patients receive the off-schedule drug in the cycling strategy.

Considerable work has built on these early studies by Bonhoeffer *et al.* [2] and Bergstrom *et al.* [3], and a range of biological features have been added to the two models. These comprise various strategies of drug adjustment in case of resistance, an explicit description of drug interactions in combination therapy and trade-offs to double resistance. Moreover, mixing and cycling with an unequal use of both drugs have been considered as well as more sophisticated strategies that monitor resistance and adjust the drug usage accordingly. The modelling framework has also been used to specifically describe treatment of gonorrhoea infection [10,14]. Table 1 gives an overview of the existing literature, including conclusions about the best treatment protocol. The table contains entries for all factors that we discuss as crucial in the following section. More details can be found in electronic supplementary material, table S1.

## 3. Challenges in the assessment of treatment strategies

In the following, we highlight and discuss a series of factors that influence the ranking of treatment strategies even though the underlying biological assumptions are (mostly) not altered. Based on the model defined in equation (2.1), we provide examples to demonstrate that these factors are indeed relevant for the ranking. For now, we do not have the information to say in which way modelling choices influence the ranking, e.g. we cannot make statements such as ‘including factor *X* into the model turns cycling into the most promising strategy’. Considering the current studies, it is not apparent that a specific choice or circumstance would systematically favour one or the other strategy (table 1).

### 3.1. The optimality criterion

**Figure 2.**
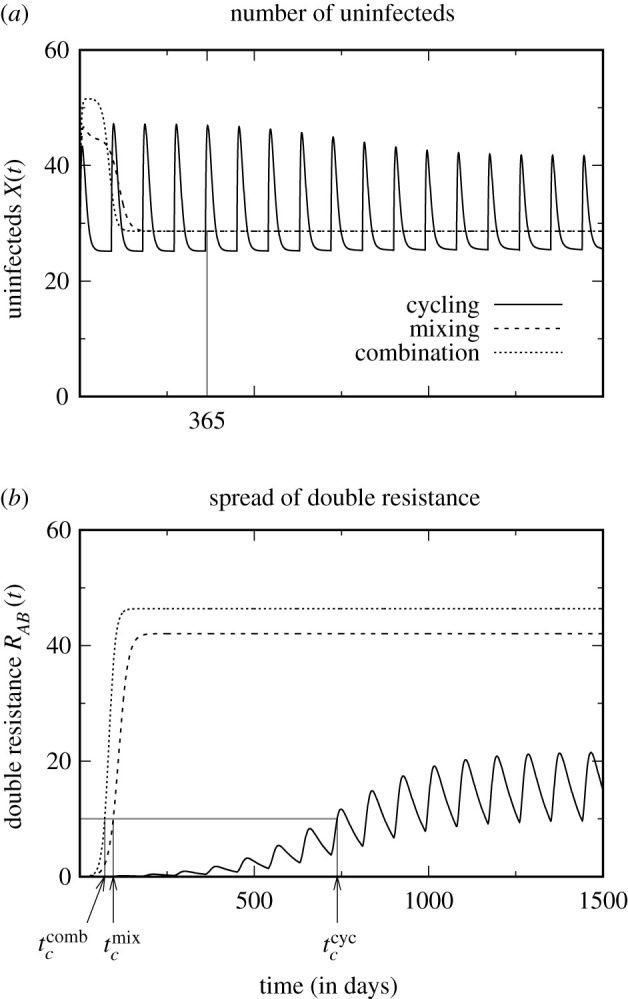
Assessment of the three treatment protocols using different optimality criteria. While the criterion *total number of uninfected patients within the first year* (*a*) ranks the strategies as ‘combination therapy > mixing > cycling’, the criteria *time until the number of patients infected with the double-resistant strain has reached 10% of all patients* and *total number of uninfected patients until this time* (*b*) lead to the ranking ‘cycling > mixing > combination therapy’. Note also that, with cycling, the double-resistant strain never reaches a very high frequency. (See electronic supplementary material for parameter values and performance scores.)

A range of optimality criteria are currently applied to assess treatment protocols. Some focus on overall treatment success (number of uninfecteds/infecteds in some time interval or at equilibrium, number of inappropriately treated patients, i.e. patients who are treated by a drug to which the infecting strain is resistant such as patients in the *R*_*A*_ compartment by drug *A*) and others on the dynamics of the resistant strains (number of patients infected with a resistant strain, emergence or spread of double resistance). As illustrated in figure 2, the choice of the optimality criterion can substantially influence the conclusions. Different treatment strategies might optimize different quantities, and a strategy might be well suited to achieve one goal but perform poorly to achieve another [16]. Hence, which criterion should be used? If the goal is to enhance our understanding of the evolutionary dynamics, any of the above listed criteria could be insightful and meaningful. If the goal is to foster clinical trials or to come to conclusions that directly guide clinical applications, answering this question is difficult. Essentially, three factors need to be taken into account: the clinical benefits (e.g. clearance of the infection) and costs (e.g. side effects) and economic costs. In any case, the modelling framework directly targets only the first of these factors. Yet, even when focusing exclusively on the clinical benefits, several aspects need to be taken into account, as discussed below. Moreover, if aiming at fostering or guiding clinical trials, one needs to choose a criterion that is suitable to serve as a clinical endpoint. Time horizons that would allow us to observe the first emergence of multiple resistance and monitor the subsequent spread of it are often not practical in clinical trials. In the discussion in the following paragraphs, we assume that sufficiently long clinical studies can be performed. However, it should be kept in mind that this may be challenging in reality, making the definition of a meaningful clinical endpoint a difficult problem, at least in situations where multiple resistant strains have not yet emerged. This emphasizes once more the importance of modelling, where no such limitations exist.

Generally, we advocate using a criterion that aims at maximizing overall treatment success rather than focusing on resistance evolution. This is because, eventually, we are not interested in the evolution of resistance *per se*—resistance could be avoided by simply not treating anybody—but rather in its harmful consequence, which is to prevent (rapid) patient recovery. Nevertheless, monitoring resistance under the various treatment strategies is by no means irrelevant for their evaluation, neither in modelling nor in clinical studies. One reason for this is that antibiotics may be abandoned when resistance reaches a threshold. That is, even if a treatment strategy reduces overall treatment failure compared with another protocol, it might lead to the earlier removal of a certain antibiotic. Given the decelerated discovery of new antibiotics, this will reduce treatment options. Moreover, the alternative antibiotic might have more severe side effects or might be substantially more expensive. *A priori*, we think that criteria that incorporate the spread of resistance are more relevant than those purely quantifying the rate of emergence. Moreover, for clinical trials, a criterion that integrates information from a time period is more robust than a criterion that relies on a single incidence or time point and that is therefore more strongly affected by stochastic effects (e.g. the first appearance of resistance).

A criterion that explicitly combines the efficacy of treatment and the spread of resistance is provided by the number of uninfected patients until the frequency of (double) resistance has reached a threshold. The disadvantage is that this entirely ignores what happens after that point in time, and this time is potentially very different for different strategies. In particular, this time is infinite in the absence of treatment, implying that treating no one achieves a perfect score, given that the number of uninfecteds at equilibrium is non-zero. Moreover, such a composite criterion makes it more difficult to understand the underlying dynamics. For clinical trials, it is again problematic since the prevalence of resistance is most likely subject to strong fluctuations, at least if measured in small units such as in a single hospital ward. Hence, the point in time at which resistance crosses the threshold is subject to stochastic variation, adding considerable extra noise and uncertainty to the data.

Two criteria that are used in a clinical context to measure the health burden of resistant infections but that have not received much attention in the mathematical literature so far are the mortality rate and the length of hospital stay (but see [16], discussed further below). Following Bergstrom *et al.* [3], many models assume that the efflux rate is independent of the infectious status, and both criteria are hence inherently meaningless in these models. Some models, in the tradition of Bonhoeffer *et al.* [2], allow for differential efflux rates of infecteds and uninfecteds. However, they do not distinguish between discharge and death (partially because they do not consider the dynamics of a hospital but of a general community, where discharge of recovereds does not occur). However, empirical therapy is especially important for critically ill and immuno-compromised patients where the bacterial infection constitutes a true burden on their health and a risk to their life. It is hence very likely that the choice of treatment—effective or ineffective—influences (i) the duration of hospitalization and (ii) the survival chances of the patient. It therefore seems highly relevant to focus on rates of discharge and death that depend on the infectious status of the patient. In this setting, the length of stay in the hospital and the mortality rate could be used as a measure of success for a treatment strategy. To assess these quantities in a deterministic framework, it is straightforward to complement the current ODE system by two further compartments, one for successfully treated and discharged (former) patients and one for the deceased. The size of the former is closely linked to the length of hospitalization; the size of the latter is closely linked to the mortality rate.

It is important to note that none of the three criteria—disease prevalence, mortality rate, length of hospitalization—is sufficient on its own but an assessment of the mortality rate should be combined with an assessment of disease prevalence or the duration of hospitalization in order to take both outcomes of inappropriate treatment (death and prolonged illness) into account. A caveat with all three criteria is that one needs to choose a time period during which their performance is assessed. At short time scales, the outcome is dominated by the transient behaviour following instalment of the new protocol. By contrast, with a long observation period, the equilibrium dynamics determine the ranking. Depending on the chosen time frame, conclusions might hence differ, and, optimally, both the short- and long-term performance should be assessed.

Since the multifacetedness of the problem does not allow us to pin down one ‘correct’ universally applicable criterion, how can we still make progress? Applying more than one criterion (ideally targeting treatment success and resistance) seems to be a sensible approach and has also been done in several studies in the past (table 1). Even if no strategy is the best under all aspects, it would be helpful to know under which criterion it is the best. This knowledge would make it possible to make an informed decision in specific cases, depending on which criterion seems to be the most important one under the given circumstances. For example, for infections where the mortality rate is high unless appropriate treatment is initiated immediately, good performance under a criterion that evaluates overall treatment success is more relevant than good performance under a criterion that focuses on the emergence of resistance. This is particularly true if a large number of antibiotics are available for this particular bacterial species. By contrast, for an infection that takes a mild course in most patients even if treated late, it might be preferable to control resistance as well as possible in order to maintain efficient treatment options with mild side effects for rare severe cases. As a side remark, to put optimality scores into context, it would be interesting to compare potential benefits of multi-drug strategies with improvements achieved by other means, such as a reduction in transmission.

### 3.2. The model implementation

#### 3.2.1. The presence or the absence of an *R*_*AB*_ compartment

**Figure 3.**
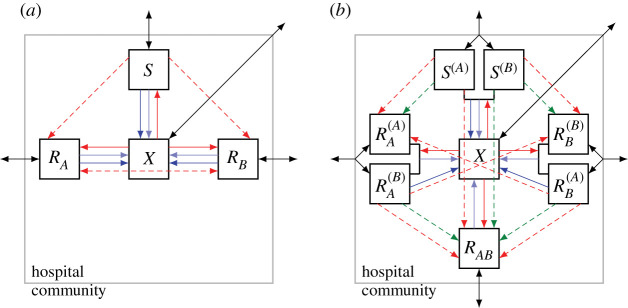
Flow diagrams of alternative model implementations. Diagram (*a*) belongs to a model without an *R*_*AB*_ compartment (no pre-existence, no influx and no *de novo* emergence of the double-resistant strain). Diagram (*b*) reflects a model where patient groups are classified not only according to the infecting strain but also according to the drug that they receive. This model, as drawn, assumes that individual patients receive the same drug throughout their entire course of treatment. Adapted from Uecker & Bonhoeffer [18].

**Figure 4.**
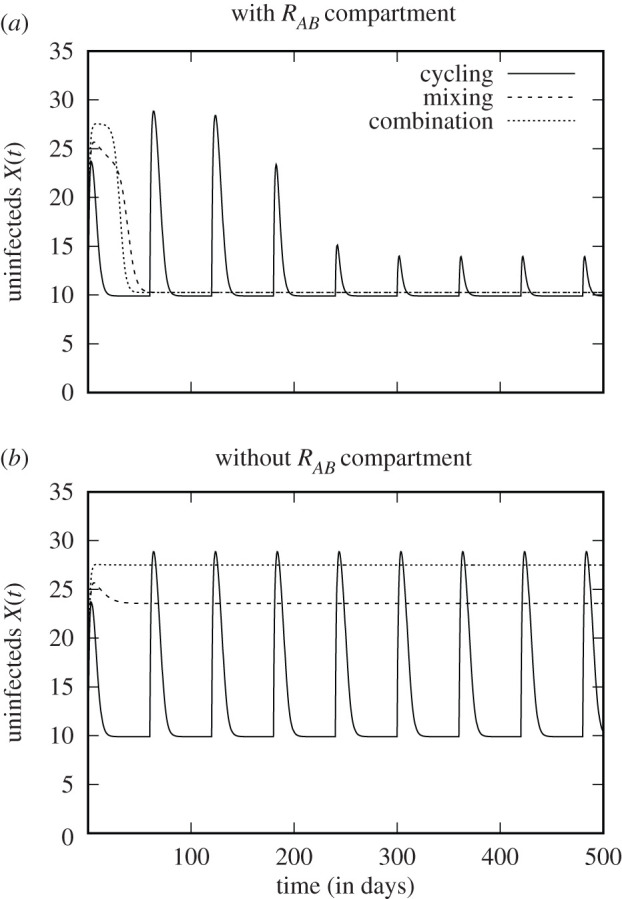
Number of uninfected patients when the model contains the *R*_*AB*_ compartment (*a*) and when it does not contain the *R*_*AB*_ compartment (*b*). For (*a*), using the number of uninfecteds within the first year as a criterion, the strategies rank ‘mixing ≳ cycling > combination therapy’. For (*b*), we obtain ‘combination therapy > mixing > cycling’. (See electronic supplementary material, for parameter values and performance scores.)

Following Bergstrom *et al.* [3], some studies use a model variant without the *R*_*AB*_ compartment (figure 3*a*). This describes the dynamics prior to the (stochastic) emergence and establishment of double resistance. Naturally, the total number of uninfecteds in a given time interval can yield a different ranking, depending on whether an *R*_*AB*_ compartment is included or not (figure 4).

In order to assess the risk of multiple resistance, studies based on this model variant consider the rate at which double resistance is generated from single-resistant strains or the sensitive strain, given by *χ*_*A*_(*t*)*ν*_*A*_*R*_*B*_(*t*) + *χ*_*B*_(*t*)*ν*_*B*_ *R*_*A*_(*t*) + *ν*_*AB*_*S*(*t*) for cycling and mixing and by *ν*_*A*_ *R*_*B*_(*t*) + *ν*_*B*_ *R*_*A*_(*t*) + *ν*_*AB*_ *S*(*t*) for combination therapy. While such an approach captures the time until the double-resistant strain first appears, it does not make any statements about how fast it will spread through the community. Protocols that delay the first appearance are not necessarily the best at slowing down its spread and vice versa. An example is shown in electronic supplementary material, figure S3.1. In this example, the double-resistant strain is generated with a higher rate under cycling than under combination therapy (and with the highest rate for mixing). However, it spreads slowest under cycling and fastest under combination therapy. This occurs because competition with the single-resistant strains hampers its frequency increase under cycling.

It is clear that excluding the possibility of double resistance from the analysis neglects a core aspect of the problem. Yet, whether the model best contains an *R*_*AB*_ compartment or not depends on the question to be answered. The *de novo* emergence of double resistance is a highly stochastic process. If the double-resistant strain is initially absent, there is a phase before it is generated (or is brought into the hospital from the outside) and starts spreading. Models without the *R*_*AB*_ compartment allow us to assess the performance of protocols during this phase and to estimate its length. Deterministic models incorporating an *R*_*AB*_ compartment ignore this phase but allow us to study the spread of double resistance.

#### 3.2.2. Deterministic versus stochastic models

The model can be implemented deterministically or stochastically. Generally, a deterministic model implementation assumes that all patient groups are large enough to neglect stochastic fluctuations. However, many models focus on treatment strategies in hospital wards, which normally only accommodate a relatively small number of patients. Even 100 patients is not a large population size if ‘large’ refers to the negligibility of stochasticity. Stochastic models are therefore *a priori* more appropriate than deterministic implementations. Especially, they may lead to different conclusions. Kouyos *et al.* [9] consider strategies where drugs do not get switched with a fixed period but in response to the frequency of resistance in the hospital. They find that this brings an advantage over mixing only when stochasticity is taken into account, while the difference disappears in a deterministic system.

Stochasticity is very high in clinical trials, making it hard to arrive at robust conclusions. Here, stochastic models can help to assess which conclusions can and cannot be drawn in the face of randomness and give a sense of the scale at which clinical studies would need to be performed. However, the role and importance of certain parameters and processes can presumably be well assessed using deterministic models.

#### 3.2.3. Model implementation with or without memory

The traditional modelling approach classifies compartments only according to the infecting strain and does not take the administered drug into account as well [18]. This approach disregards the associations that build up between the infecting strain and the drug used. (These associations build up since patients treated with the wrong drug recover more slowly.) This simplified model is often a good approximation, leading to similar predictions as a model that classifies patients by both the infecting strain and the drug used (figure 3*b*). However, awareness of the simplification seems important. Instead of considering it as a simplification, the traditional modelling choice can also be explained by a different interpretation of the parameter *τ*, as done in several studies. These studies assume that patients get treated at rate *τ* and, upon treatment, recovery is instantaneous. Again, it is important to account for the meaning of *τ* in the models when reading the results.

### 3.3. The model analysis

The models are challenging to analyse for two reasons. First, owing to the model complexity, most studies rely on numerical simulations rather than on analytical approximations. It is hence not possible to read off general results from an analytical solution. Second, the parameter space is very large and parameter estimates are lacking. Trivially, all conclusions from numerical simulations are *a priori* only valid for the chosen parameter sets (e.g. electronic supplementary material, figure S3.3). It has been less appreciated that not only the chosen parameter values but also the initial conditions for the ODE system (equation (2.1)) may influence the relative ranking of treatment protocols, since the number of patients in each compartment at time *t* = 0 influences the early dynamics (electronic supplementary material, figure S3.4).

Parameter sensitivity and structural sensitivity tests can alleviate the problem (see Tepekule *et al.* [17] for a study that implements random sampling of parameters, linear discriminant analysis and particle swarm optimization to systematically explore the parameter space and that also investigates the influence of the initial conditions; for an insightful discussion of structural sensitivity in between-host models of antibiotic resistance, see Spicknall *et al.* [29]). Importantly, this includes allowing for asymmetry between the two single-resistant strains and for unequal use of the two drugs, e.g. a drug ratio other than 50 : 50 for the mixing strategy [5]. It is also important to investigate by how much strategies differ from each other (and from mono-drug therapies, which sometimes even outperform multi-drug strategies, [17]). If differences are small, a ranking may be meaningless.

For the ranking of strategies, a numerical analysis is required to be able to allow for sufficient model complexity. For a more fundamental understanding of the model behaviour, e.g. for understanding the reasons why a strategy performs better or worse than expected, an analytical treatment of simplified models or limiting cases may provide further insight.

## 4. Important model extensions

The modelling framework allows, of course, for innumerous extensions but we will only discuss two potential directions here.

Nested models that describe both the within-host dynamics of the bacteria and the epidemiological dynamics could help to gain a more realistic picture. In the current epidemiological models, the transition rates (recovery rate, emergence of resistance, etc.) are modelled by independent parameters. However, these rates are in reality coupled and explicit modelling of the within-host dynamics would account for these inter-dependencies. Of course, the approach would require us to make assumptions about the parameters at the within-host level that are equally unknown as the parameters at the between-host level, hence leading to considerable uncertainty about the appropriate model and its parametrization. Nested models should therefore not replace but complement the current simpler models. To our knowledge, Beardmore *et al.* [16] is the only study that has implemented a nested model in this context so far. In a briefly presented model, the bacterial load within individual patients is tracked, and discharge of patients depends on their bacterial load, making the length of hospital stay variable (there is no mortality in the model). The duration of hospitalization is used to assess the performance of the two treatment strategies, cycling and mixing. Further development of models along these lines could greatly enhance our understanding of the prospects of antibiotic treatment protocols.

Last, nosocomial infections are often caused by commensal bacteria. They may either stem from the patient’s own flora and may have already been present at admission or be acquired asymptomatically within the hospital. Or they may be acquired as pathogens from other patients. These different routes of infection and ways of transmission are not accounted for by most modelling studies. Most studies follow the approach of traditional epidemic models for the spread of obligate pathogens. For example, transition from the *X* to any infected compartment is only possible through infection from infected patients rather than through self-infection from own commensals. Rethinking the models in the context of commensal bacteria as agents of infection seems to be important in order to build a framework that is fully consistent with its objectives. In this context especially, the consequences of horizontal gene transfer should be considered. Clinically relevant resistance is often encoded on plasmids. Yet, how this influences the effectiveness of cycling, mixing or combination therapy has only been touched upon [3].

## 5. Concluding remarks

Which strategy is optimal is determined by the interplay of many factors, making general answers difficult. Yet, systematic approaches can at least allow us to make probabilistic statements. For example, Tepekule *et al.* [17] determined how likely each strategy is to ‘win’ across the parameter range and identified parameter regimes for which combination therapy is particularly likely to fail. By performing similar analyses for different optimality criteria or model implementations and a comparison of results, we could learn more about the conditions under which any strategy is particularly good or bad. It would then also be important to assess how robust the results are with respect to imperfections in the implementation of strategies: in reality, treatment decisions are based on the medical condition of the individual patient, and it will never be possible to treat each single patient according to a hospital-wide protocol.

Even if we might have preferred to identify one strategy as universally the best, the complex picture that has emerged from mathematical models so far should be appreciated as a result in its own right. It should also be appreciated that it is the result of a scientific discourse and a development brought forward by a series of articles. It was by no means clear *a priori* that no simple answer exists.

The focus of this article is on the role of mathematical models in the assessment of antibiotic treatment protocols and on ways to improve their contribution. However, a joint effort is necessary to arrive at good solutions. The theoretical work reviewed in this article is not discussed here in the context of clinical studies, while such a connection could lead to more potent studies. This would be particularly valuable if, through sequencing and the analysis of sequence data, the pathway of resistance could be traced back along patients in order to distinguish *de novo* acquired from transmitted resistance. Besides models and clinical studies, a third tool, which has surprisingly been understudied so far, is *in vitro* experimental evolution. While evolution experiments are widely used to investigate the evolution and maintenance of antibiotic resistance and are also used to study the effect of combining antibiotics, population-wide treatment strategies have to our knowledge barely been simulated in the laboratory (for a recent exception, see [30]). As an intermediate between models and clinical trials, they can help to close the gap between theoretical and clinical studies in the future.

## Data accessibility

The Mathematica notebooks used to generate the data for the figures are available as electronic supplementary material.
